# Isoniazid Prophylactic Therapy for the Prevention of Tuberculosis in HIV Infected Adults: A Systematic Review and Meta-Analysis of Randomized Trials

**DOI:** 10.1371/journal.pone.0142290

**Published:** 2015-11-09

**Authors:** Henok Tadesse Ayele, Maaike S. M. van Mourik, Thomas P. A. Debray, Marc J. M. Bonten

**Affiliations:** 1 Department of Public Health, College of Health Sciences & Referral Hospital, Dilla University, Dilla, Gedeo Zone, Ethiopia; 2 Department of Infectious Diseases’ Epidemiology, Julius Center for Health Sciences & Primary Care, University Medical Center Utrecht, Utrecht, The Netherlands; 3 Department of Epidemiology, Julius Center for Health Sciences & Primary Care, University Medical Center Utrecht, Utrecht, The Netherlands; 4 Department of Medical Microbiology and Infection Control, University Medical Center Utrecht, Utrecht, The Netherlands; University of Cape Town, SOUTH AFRICA

## Abstract

**Background:**

Infection with Human Immunodeficiency virus (HIV) is an important risk factor for Tuberculosis (TB). Anti-Retroviral Therapy (ART) has improved the prognosis of HIV and reduced the risk of TB infected patients. Isoniazid Preventive Therapy (IPT) aims to reduce the development of active TB in patients with latent TB.

**Objective:**

Systematically review and synthesize effect estimates of IPT for TB prevention in adult HIV patients. Secondary objectives were to assess the effect of IPT on HIV disease progression, all-cause mortality and adverse drug reaction (ADR).

**Search Strategy:**

Electronic databases were searched to identify relevant articles in English available by September 11^th^ 2015.

**Selection Criteria:**

Research articles comparing IPT to placebo or no treatment in HIV infected adults using randomized clinical trials.

**Data Analysis:**

A qualitative review included study-level information on randomization and treatment allocation. Effect estimates were pooled using random-effects models to account for between-study heterogeneity.

**Main Results:**

This review assessed ten randomized clinical trials that assigned 7619 HIV patients to IPT or placebo. An overall 35% of TB risk reduction (RR = 0.65, 95% CI (0.51, 0.84)) was found in all participants, however, larger benefit of IPT was observed in Tuberculin Skin Test (TST) positive participants, with pooled relative risk reduction of 52% [RR = 0.48; 95% CI (0.29, 0.82)] and with a prediction interval ranging from 0.13 to 1.81. There was no statistically significant effect of IPT on TB occurrence in TST negative or unknown participants. IPT also reduced the risk of HIV disease progression in all participants (RR = 0.69; 95% CI (0.48, 0.99)) despite no benefits observed in TST strata. All-cause mortality was not affected by IPT although participants who had 12 months of IPT tend to have a reduced risk (RR = 0.65; 95% CI(0.47, 0.90)). IPT had an elevated, yet statistically non-significant, risk of adverse drug reaction [RR = 1.20; 95% CI (1.20, 1.71)]. Only a single study assessed the effect of IPT in combination with ART in preventing TB and occurrence of multi-drug resistant tuberculosis.

**Conclusions:**

IPT use substantially contributes in preventing TB in persons with HIV in general and in TST positive individuals in particular. More evidence is needed to explain discrepancies in the protective effect of IPT in these individuals.

## Introduction

*Mycobacterium tuberculosis* (TB) is the most common cause of bacterial infection in humans[1–3] and is globally a leading cause of morbidity and mortality, especially in developing countries.[4] Human Immunodeficiency Virus (HIV) infection is the strongest risk factor for TB and over 4 million people are co-infected with both organisms, the majority of whom reside in Africa.[5] Such co-infection worsens the prognosis of HIV infection by increasing HIV replication[6–8] and may result in rapid progression of HIV and subsequent immunosuppression,[9–12] and a higher risk of acquiring other, potentially lethal, opportunistic infections.[13], [14]

Randomized controlled trials have demonstrated that a course of Isoniazid Preventive Therapy (IPT) reduces the incidence of TB disease in HIV-negative populations at risk of developing active disease.[15] In HIV infected patients, IPT reduced reactivation of latent TB infection, both in industrialized countries[16–18] as well as in developing countries.[19–21] Also, observational studies in HIV-positive injecting drug users (IDU) suggested a potential benefit of IPT.[16], [17], [22–24]. The benefit appears to be higher in Tuberculin Skin Test (TST) positive patients than in TST negatives.[25] In addition, recent observational studies reported that the benefit of IPT increased if it is delivered in combination to ART.[26–28]

A previous meta-analysis found that IPT was efficacious in TST positive participants in reducing risk of TB.[29–31] Based on these data, the World Health Organization (WHO) in 2004 recommended IPT for HIV-infected persons[32–34] and most high TB/HIV burden countries have adopted antiretroviral therapy (ART) and the WHO recommended TB/HIV collaborative activities including IPT since 2005. However, previous meta-analyses did not include studies with participants on ART nor assessed the effect of IPT in different dose and duration strata.[35] In addition, the recently updated WHO guidelines[36] does not precisely outline the optimal dosing and duration of IPT treatment in patients receiving ART.[30]

This study aims to synthesize evidence from randomized controlled trials regarding the protective effect of IPT on all-type or confirmed TB in HIV infected patients, and assess the combined benefit of ART an IPT on prevention of active TB. Secondary objectives were to assess the effect of IPT on HIV disease progression, all-cause mortality and adverse drug reaction (ADR) as well as to determine the effect of IPT in strata of TST positive and negative patients, and by dosing and duration of the IPT regimen.

## Methods

### Eligibility and selection

This systematic review included clinical trials assessing the effect of IPT on prevention of active TB or reactivation of latent TB disease in adults living with HIV with or without ART. Studies were eligible when the intervention (IPT) was applied to adult HIV patients (excluding pregnant women), had at least one year follow-up after finishing IPT and used a randomized study design. Furthermore, reported data included the occurrence of active TB disease.

### Search methods for the identification of studies

A comprehensive search of PubMed, Excerpta Medica dataBASE (EMBASE), the Cochrane Central Register of Controlled Trials (CENTRAL) and Cumulative Index of Nursing and Allied Health (CINAHL) was performed to identify all relevant studies in the English language available from the start of MEDLINE to September 11^th^ 2015. Both text words in title, abstract and medical subject heading (MeSH) terms were used in varying combinations. The literature search strategy was adapted to suit each database (Table 1). Moreover, the website of Health Internet Internetwork and Research Initiative (HINARI) and Google Scholar databases were checked for potential eligible studies.

**Table 1:** Search strategy.

	Domain, intervention, outcome and design			
	HIV/AIDS (1)	Isoniazid (2)	Tuberculosis (3)	Randomization (4)
Search terms	Human immunodeficiency **OR** virus, **OR** HIV, **OR** Acquired immunodeficiency syndrome, **OR** AIDS	Isoniazid, INH, **OR** Prevention, **OR** Preventive, **OR** Prophylaxis, **OR** Prophylactic	Tuberculosis **OR** TB	Randomized **OR** Randomization
Search fields	MeSH **AND** Title/Abstract	MeSH **AND** Title/Abstract	MeSH **AND** Title/Abstract	MeSH **AND** Title/Abstract
Final search	(((#1) **AND** #2) **AND** #3) AND #4		Date of search closure at September 11^th^ 2015	

**HIV**: Human Immunodeficiency Virus; **AIDS**: Acquired Immunodeficiency Syndrome; **INH**: Isoniazid; **TB**: Tuberculosis; **MeSH**: Medical Subject Headings

### Data collection

A pre-defined data extraction tool was used to extract information on IPT and outcome related variables. Details on the intervention included the regimen of the preventive therapy, dosing, duration, randomization processes, follow up periods, and combined use of IPT and ART. In addition, study area, publication year, and methods of outcome ascertainment were extracted. (S1 Table) We assessed comparability of the target population, intervention and outcome across the selected studies.

### Outcome

The primary outcome of interest was the onset of active TB disease. The ascertainment was mostly based on detection of acid fast bacilli (AFB), pathology, clinical features and X-ray (all-types of TB) or confirmed by culture positivity of sputum or biopsy (confirmed TB). In addition, HIV disease progression, all-cause mortality and ADR which caused withdrawal of patients from the follow-up were considered as secondary end points.

### Determinant

The primary determinant was IPT stratified by TST status. TST reactivity demonstrates a functional anti-mycobacterial response; a TST negative status can therefore indicate a lack of TB exposure, but also result from severe immune deficiency (anergy) with or without underlying latent TB. A TST positive status, therefore, demonstrates TB exposure as well as a preserved immune response. The latter could affect the efficacy of IPT. Analyses were further stratified by dosage and duration of the IPT regimen.

### Statistical Methods

The reviewed articles’ unit of analysis was the individual patient. All the randomization and intervention allocation was patient-based. Due to the underlying clinical heterogeneity of selected studies, a random-effects model was used to estimate the pooled effect of IPT, using the method of Hartung and Knapp.[37], [38] The between-study heterogeneity in treatment effect was assessed by the I squared (I^2^) statistic and 95% prediction intervals (95% PI). The I^2^ statistic indicates the extent of variability that cannot be explained by sampling error and ranges between 0 and 100%. Conversely, the prediction interval gives an indication of true treatment effect of IPT that can be expected when the intervention is applied to new studies; it thereby provides a sense of the heterogeneity in expected benefits.[39] Furthermore, subgroup analyses were performed and stratified by baseline TST status (positive, negative, and unknown) and dosing and duration of the regimen. MetaAnalyst[40] and R version 3.2.1[41] were used for the data analysis.

## Results

### Study selection

**Fig 1:**
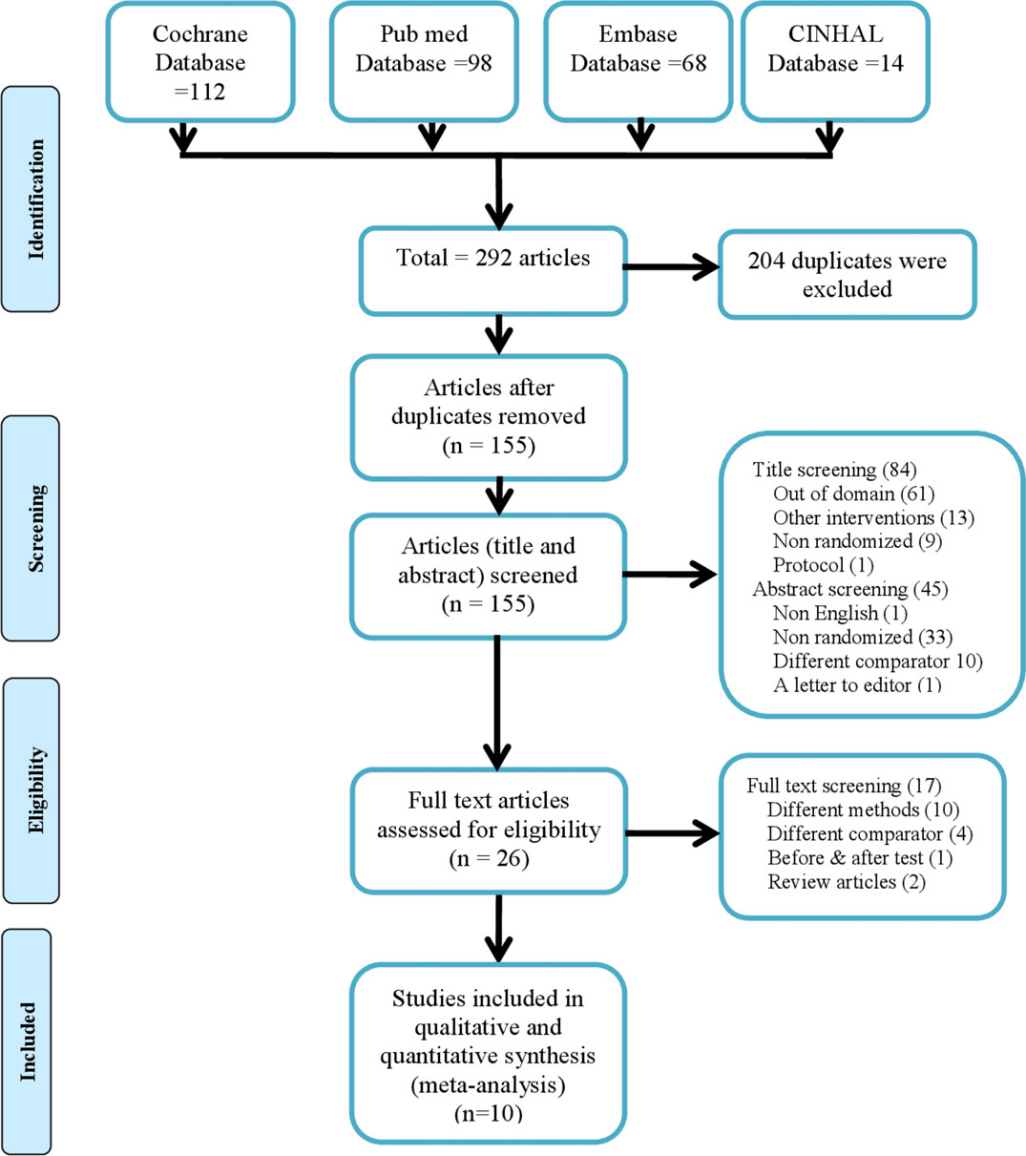
Schematic presentation of studies inclusion to the review.

Based on the preset inclusion and exclusion criteria and cross referencing, ten articles were eligible for inclusion in this review from 246 initial hits from four databases. (S1 Text and S2 Text) After de-duplication, 117 studies underwent application of in- and exclusion criteria, leaving ten articles for this meta-analysis. (Fig 1)

### Description of included studies

Of the included studies, eight were conducted in six high TB/HIV burden countries (Uganda, Cote d’Ivoire, Kenya, South Africa (2), Zambia, and Haiti (2))[42] and two were in countries with low TB endemicity (Spain and United States of America (USA)). Table 2 provides details on study characteristics.

**Table 2:** Description of selected studies for the systematic review and meta-analysis.

Study population				Exposure						Outcome			Analysis	
Studies	n	Age	TST status	ART use	IPT regimen	Placebo regimen	Duration of follow-up	Method of randomi-zation	Blinding	Ascertainment of TB	Lost to follow-up	Adherence	Other endpoints	Analysis
Fitzgerald 2001 (Haiti)	237	≥ 18	Negative	No	IPT 300 mg + VB6 50 mg daily (12 mo)	VB6 50 mg for (12 mo)	2.5 years in IPT & 2.4 years in placebo	Unclear	Not clear	Clinical, AFB, Radiology	23% lost to follow up	No data	all-cause mortality & AIDS	ITT
Gordin 1997 (USA)	517	≥ 13	Negative	No	IPT 300 mg + VB6 50 mg daily (6 mo)	Placebo + VB6 50 mg daily for (6 mo)	30 months	Unclear	Not clear	Culture, AFB, Radiology, Clinical	6.2% (IPT), 7% (placebo)	37% non-adherence	Probable TB, all-cause mortality, AIDS	Not mentioned (PP assumed)
Hawken 1997 (Kenya)	684	14–65	Positive & negative	No	IPT 300 mg daily (6 mo)	Placebo daily (6 mo)	Median 22 months (0–41 months)	Blocked (per 10)	Double blinded	Culture & Histology	29.8% (at 6 months)	42% missed 1 week, 27% missed 1 – 4wk and 31% missed > = 5	AIDS, all-cause mortality, ADR	Not mention-ed (PP assumed)
Moham-med 2007 (South Africa)	98	≥ 18	Negative No	No	IPT 15 mg/kg + VB6 25mg twice weekly (12 mo)	Placebo + VB6 25mg twice weekly (12 mo)	12 months	Blocked (per 20)	Double blinded	Culture-positive, Radiographic, & AFB	Complete	15% non-adherence	All-cause mortality, Adherence, ADR	ITT
Mwinga 1998 (Zambia)	1053	≥ 15	Positive & negative	No	IPT 900 mg twice weekly (6 mo)	Placebo twice weekly (6 mo)	36 months	Blocked (per 30)	Double blinded	AFB, Radiology & Culture	32.4% (IPT), 30.3% (placebo)	Non-adherence 28% for IPT & 18% for placebo	All-cause mortality & ADR	ITT
Pape 1993 (Haiti)	118	18–65	positive & negative	No	IPT 300 mg + VB6 50 mg daily (12 mo)	VB6 50 mg daily (12 mo)	Placebo 2.45 years & IPT 3.13 years	Computer generated	Double blinded assumed	AFB, Radiology, Histopatho-logy, Culture, Clinical	Unclear	Unclear	AIDS & all-cause mortality	ITT
Rangaka (2014) (South Africa)	1329	≥ 18	Both TST & IGRA positive & negative	Both at ART at base-line	IPT (<50kg 200 mg or ≥50kg 300mg) daily and VB6 (12 mo)	Placebo daily for (6 mo)	>2.5 years (maximum 3.7 years)	Random number generator	Double blinded	AFB, Culture and Histo-pathology	11%	No data	All-cause mortality & ADR	Modified ITT
Rivero 2003 (Spain)	319	18–65	Negative	No	IPT 5mg/kg (300mg max) daily for 6 months	No IPT	2 years	Unclear	Open label	AFB, Culture and clinical	6.1% (IPT)	79.3% (IPT)	All-cause mortality and ADR	Not mention-ed (PP assumed)
TEMPRANO 2015	2056	≥ 18	Both IGRA positive & negativeNo TST	Early versus deferred ART	IPT 300 mg for six months	No IPT	30 months	Stratified, computer generated, sequentially numbered, & bock randomization	Open label	AFB, Culture and clinical	2.2% (IPT) 3.4% (No IPT)	Deferred IPT 93% Early IPT 94%	Grade III or IV illness, Virologic suppression, & Adherence	ITT
Whalen 1997 (Uganda)	2736	≥ 18	positive & negative (Anergy)	ART excluded	IPT 300 mg daily (6 mo)	Vit C 250 mg daily (6 mo)	15 months	Blocked (per 6)	Blinded	AFB & Culture	12% TST positive & 14% TST negatives	No data	All-cause mortality & ADR	ITT

**ADR:** Adverse Drug Reaction; **AFB:** Acid fast bacilli; **AIDS:** Acquired immune deficiency Syndrome; **ART:** Antiretroviral Therapy; **IGRA:** Interferon Gamma Release Assay; **IPT:** Isoniazid Preventive Therapy; **ITT:** Intention To Treat; **LTF:** Lost To Follow-up; **PP:** Per Protocol; **TB:** Tuberculosis; **TST:** Tuberculin Skin Test; **VB6:** Vitamin B 6 (Pyridoxine)

All studies randomized participants to IPT or placebo or no IPT intervention; however, the dosage and duration of IPT varied. The total duration of the IPT treatment course ranged from 6 to 12 months with subsequent follow up of 12–44 months. Most of the included studies (n = 7) assessed 300mg of IPT daily for a duration of six months (n = 4) or twelve months (n = 3). A Zambian study assessed 900 mg of IPT twice weekly for 12 months and another South African study assessed 15mg/kg of IPT twice weekly. Six studies assessed the effect of Isoniazid alone in comparison to placebo or no intervention whereas two studies assessed the effect of Isoniazid alone compared to combinations of Rifampicin and Pyrazinamide with placebo or no intervention. The recent TEMPRANO study compared IPT treatment effect to no IPT in the setting of deferred ART or early ART.

With the exception of two studies, the included studies assessed the TST status at baseline. Four studies included both TST positive and negative individuals while three studies solely considered TST negatives. The Ugandan study separately investigated the effect of IPT in TST positive and anergic patients. In most African countries ART was widely introduced in 2004 and five studies conducted before this period lack information regarding ART use. Five studies used a block randomization, two studies used computer generated randomization and the remaining two studies used unclear randomization methods.

### Methodological quality assessment

The qualitative assessment on data completion, randomization, treatment concealment and outcome blinding did not reveal major methodological flaws across studies. In some studies, exclusion of possible pre-existing TB disease by ascertainment of clinical symptoms and signs at baseline may have missed few cases of pre-existing TB. Ascertainment of the outcome of TB was active and mostly based on AFB test confirmed by culture positivity of sputum or biopsy. Most of the included studies used family-reported all-cause mortality. There was no indication of selective reporting[43] (Table 3).

**Table 3:** Risk of bias summary of included studies.

Studies	Fitzgerald 2001	Gordin 1997	Hawken 1997	Mohammed 2007	Mwinga 1998	Pape 1993	Rangaka 2014	Rivero 2003	TEMPRANO 2015	Whalen 1997
Optimal exclusion of TB patients at enrolment	**?**	**+**	**+**	**+**	**+**	**+**	**?**	**+**	**+**	**+**
Random sequence generation	**+**	**+**	**+**	**+**	**+**	**+**	**+**	**+**	**+**	**+**
Allocation concealment	**-**	**+**	**+**	**+**	**+**	**-**	**+**	**?**	**-**	**+**
Placebo control	**+**	**+**	**+**	**+**	**+**	**+**	**+**	**-**	**-**	**+**
Blinding of participants, personnel, and outcome assessors (TB, ADR, and AIDS)	**?**	**+**	**?**	**+**	**+**	**-**	**+**	**?**	**-**	**+**
Completion of IPT course with adequate adherence	**?**	**-**	**+**	**+**	**-**	**+**	**+**	**-**	**+**	**+**
Lost to follow up described	**+**	**+**	**+**	**+**	**+**	**+**	**+**	**+**	**+**	**+**
Both primary and secondary outcomes were reported	**+**	**+**	**+**	**+**	**+**	**+**	**+**	**+**	**+**	**+**

**TB**: Tuberculosis; **ADR**: Adverse Drug Reaction; **AIDS**: Acquired Immuno Deficiency Syndrome; **IPT**: Isoniazid Preventive Therapy

**+** Low risk of bias

**?** Unclear (probable) risk of bias

- High risk of bias

### Assessment of heterogeneity

Among the included studies, there was substantial clinical and methodological variability, complicating interpretation of study findings. The clinical heterogeneity emanates in part from baseline variations of subjects such as TST status variation. In addition, the IPT intervention differed in terms of dose, duration, provision of IPT with other preventive therapies, adherence levels achieved and subsequent follow-up. Other methodological variation across studies was mostly related to differences in randomization techniques, outcome ascertainment and analysis methods. Finally, eight studies were conducted in low income and high TB burden countries; however, the other two studies were conducted in a high income country with lower levels of endemicity. Given these differences, it is recommended to allow for the between-study heterogeneity in treatment effect and to adopt random effects models.[37], [38], [44] Despite the presence of clinical heterogeneity between the included studies, an acceptable degree of statistical heterogeneity was found for the analysis including all relevant studies. A somewhat larger statistical heterogeneity was observed in the subset of studies where IPT effect on all-cause mortality was assessed in TST positive participants (I^2^ = 54.7%) and IPT effect on HIV disease progression (I^2^ = 59.4%). Prediction intervals were determined to indicate an anticipated variation in effect estimates.

### Effects of IPT

#### Overall population

IPT showed significantly reduced relative risk (RR) of TB (RR = 0.65 with 95% CI: 0.51, 0.84), substantial heterogeneity was however present in this effect (95% PI: 0.37, 1.17) (Table 4) Similar results were found for the effect of IPT toward confirmed TB (RR = 0.69, 95% CI: 0.48, 0.99) with a prediction interval (95%) of 0.38 to 1.24) (Table 5). IPT tended to reduce the risk of all-cause mortality yet statistically non-significant RR = 0.90(95% CI (0.79, 1.02) and 95% PI (0.71, 1.13)) (Table 6). The effect of IPT was almost null on risk of HIV disease progression RR = 0.99; 95% CI (0.73, 1.34) and wide 95% PI (0.31, 3.18). (Table 7)

**Table 4:** The pooled estimates of isoniazid preventive therapy effect on all-types of Tuberculosis.

Exposure category		Number of studies	Sample size	Pooled RR* (95% CI)	95% PI
Overall estimate		10	7619	0.65 (0.51, 0.84)	(0.37, 1.17)
TST	Positive	5	1703	0.48 (0.29, 0.82)	(0.13, 1.81)
	Negative	9	3140	0.79 (0.58, 1.08)	(0.54, 1.16)
	Unknown	4	2776	0.68 (0.42, 1.10)	(0.11, 4.23)
ART	Treated	2	2226	0.67(0.47, 0.96)	Not estimable**
	Not treated	8	4234	0.73 (0.53, 1.02)	(0.33, 1.60)
IPT dose	300mg	8	6819	0.62 (0.47, 0.82)	(0.34, 1.12)
	900mg	2	800	0.89 (0.36, 2.18)	Not estimable**
IPT duration	6 months	6	5837	0.61 (0.45, 0.82)	(0.30, 1.22)
	12 months	4	1782	0.79 (0.45, 1.37)	(0.11, 5.73)

* *Random effect model*

**Prediction interval can only be estimated for more than two studies

**ART**: Antiretro viral therapy; **CI**: Confidence interval; **IPT**: Isoniazid preventive therapy; **PI**: Prediction interval; **RR**: Relative Risk; **TST**: Tuberculin Skin Test

**Table 5:** The pooled estimates of isoniazid preventive therapy effect on confirmed Tuberculosis.

Exposure category		Number of studies	Sample size	Pooled RR* (95% CI)	95% PI
Overall estimates		5	3392	0.69 (0.48, 0.99)	(0.38, 1.24)
TST	Positive	1	112	0.13 (0.01, 2.32)	Not estimable**
	Negative	3	1021	0.77 (0.36, 1.64)	(0.01, 106.22)
	Unknown	2	930	0.82 (0.47, 1.43)	Not estimable**

* Random effect model

**Prediction interval can only be estimated for more than two studies

**CI**: Confidence interval; **IPT**: Isoniazid preventive therapy; **PI**: Prediction interval; **RR**: Relative Risk; **TST**: Tuberculin Skin Test

**Table 6:** The pooled estimates of isoniazid preventive therapy effect on all-cause mortality.

Exposure category		Number of studies	Sample size	Pooled RR* (95% CI)	95% PI
Overall estimates		10	7657	0.90 (0.79, 1.02)	(0.71, 1.13)
TST	Positive	4	1311	0.67 (0.34, 1.35)	(0.04, 10.10)
	Negative	7	2428	1.02 (0.90, 1.15)	(0.86, 1.20)
	Unknown	3	2400	0.74 (0.52, 1.05)	(0.08, 7.19)
ART	Treated	2	2226	0.77 (0.46, 1.28)	Not estimable**
	Not treated	8	4272	0.92 (0.81, 1.05)	(0.72, 1.18)
IPT dose	300mg	8	6819	0.91 (0.80, 1.03)	(0.73, 1.13)
	900mg	2	838	0.81 (0.46, 1.40)	Not estimable**
IPT duration	6 months	6	5855	0.95 (0.85, 1.07)	(0.81, 1.12)
	12 months	4	1802	0.65 (0.47, 0.90)	(0.32, 1.32)

* Random effect model

**Prediction interval can only be estimated for more than two studies

**ART**: Antiretro viral therapy; **CI**: Confidence interval; **IPT**: Isoniazid preventive therapy; **PI**: Prediction interval; **RR**: Relative Risk; **TST**: Tuberculin Skin Test

**Table 7:** The pooled estimates of isoniazid preventive therapy effect on HIV disease progression.

Exposure category		Number of studies	Sample size	Pooled RR* (95% CI)	95% PI
Overall estimates		4	4929	0.99 (0.73, 1.34)	(0.31, 3.18)
TST	Positive	1	63	0.36 (0.15, 0.85)	Not estimable**
	Negative	3	809	1.00 (0.88, 1.15)	(0.42, 2.40)
	Unknown	1	2056	1.38 (0.79, 2.40)	Not estimable**

* Random effect model

**Prediction interval can only be estimated for more than two studies

**CI**: Confidence interval; **PI**: Prediction interval; **RR**: Relative Risk; **TST**: Tuberculin Skin Test

#### TST positive

In TST positive participants, five studies including a total of 1703 participants demonstrated an effect of IPT on all types of TB (probable to confirmed) with a pooled RR of 0.48; (95%CI 0.29–0.82) and 95% PI ranging from 0.13 to 1.81. Only one study reported the results for confirmed TB [RR = 0.13; 95% CI (0.01, 2.32)]. Four studies, including 1311 participants, reported the effect of IPT on all-cause mortality with pooled RR of 0.67; 95% CI (0.34, 1.35) with a wide prediction interval of (0.04, 10.10). Data about the effect of IPT on HIV disease progression was could be derived from one study only (RR = 0.36; 95% CI (0.15, 0.85)). (Tables 4–7)

#### TST negative

Nine studies reported data for TST negative (n = 3140) participants. The pooled relative risk for IPT on all types of TB was 0.79 (95% CI: 0.58, 1.08) with a prediction interval of (0.54, 1.16). For confirmed TB only, data was obtained from three studies including 1021 participants. The pooled RR was 0.77 (95% CI: 0.36, 1.64). All-cause mortality was determined from seven studies with 2428 participants. The pooled RR of IPT on all cause-mortality was 1.02 (95% CI: 0.90, 1.15) with a prediction interval of (95% PI: 0.86, 1.1.20). Three studies with 809 participants could deliver data about HIV disease progression, RR = 1.00; 95% CI (0.88, 1.15) with a prediction interval of from 0.42 to 2.40. (Tables 4–7)

#### TST unknown

In participants with an unknown TST status, the pooled relative risk of IPT on all types of TB was 0.68; 95% CI (0.42, 1.10) with a very wide prediction interval of (95% PI: 0.11, 4.23). Based on two studies with 930 participants, the pooled RR of IPT on confirmed TB was 0.82; 95% CI (0.47, 1.43). Similarly, the other three studies had data on all-cause mortality and the pooled RR was 0.74; 95% CI (0.52, 1.05). One study with 2056 participants found an elevated risk of HIV disease progression yet statistically non-significant RR = 1.38; 95% CI (0.79, 2.40).

#### ART treatment

IPT-ART combination treatment could be estimated from two studies with 2226 participants. IPT reduced risk of all-types of TB in participants treated with ART RR = 0.67 (95% CI 0.47, 0.96). The pooled effect from studies (n = 8) which did not include participants on ART was not statistically significant RR = 0.73; 95% CI (0.53, 1.02) and 95% PI (0.33, 1.60). In the same studies, the pooled effect of IPT on all-cause mortality for participants on ART was 0.77; 95% CI (0.46, 1.28) and 0.92; 95% CI (0.81, 1.05) for those not on ART. (Tables 4, 6 & 8)

**Table 8:** The pooled estimates of isoniazid preventive therapy effect on adverse drug reaction.

Exposure category		Number of studies	Sample size	Pooled RR* 95% CI	95% PI
Overall estimates		9	7284	1.20 (0.85, 1.71)	(0.50, 2.88)
ART	Treated	2	2226	1.41 (0.81, 1.71)	Not estimable**
	Not treated	7	3899	2.06 (0.96, 4.40)	(0.24, 17.88)
IPT dose	300mg	8	6582	1.06 (0.79, 1.41)	(0.54, 2.10)
	900mg	1	702	3.98 (1.13, 13.97)	Not estimable**
IPT duration	6 months	7	5837	1.47 (0.83, 2.60)	(0.31, 6.94)
	12 months	2	1447	1.09 (0.84, 1.42)	Not estimable**

* Random effect model

**Prediction interval can only be estimated for more than two studies

**ART**: Antiretro viral therapy; **CI**: Confidence interval; **IPT**: Isoniazid preventive therapy; **PI**: Prediction interval; **RR**: Relative Risk; **TST**: Tuberculin Skin Test

#### IPT dose and duration

The 300 mg IPT daily dose had higher benefit than 900mg twice weekly dose in reducing all-types of TB risk, pooled RR = 0.62; 95% CI(0.47, 0.82) and 0.89; 95% (0.36, 2.18) respectively. There were no differences in the pooled effect of different IPT doses on risk of all-cause mortality. Concerning the duration of IPT, 6 months therapy reduced all-types of TB [RR = 0.61; 95% CI (0.45, 0.82)]. The same protective effect of 12 months IPT course observed toward all-types of TB risk reduction yet the pooled estimate was not statistically significant [RR = 0.79; 95% CI (0.45, 1.37)] with a wide 95% PI (0.11, 5.73). (Tables 4, 6 & 8)

For adverse drug reactions participants were not stratified by TST status. All studies included data about the overall number of ADR (5068 participants). Nevertheless, two studies did not have withdrawal from the follow up due to ADR. The pooled RR of IPT compared to placebo for the development of severed ADR was 1.32; 95% CI (0.89, 1.96) with prediction interval of (0.46, 3.75). For the included studies, there appeared a positive association between the dosage of IPT and the risk of adverse drug reaction. The 900mg IPT dose showed higher risks of ADR [RR = 3.98; 95% CI (1.13, 13.97)] in comparison to 300mg daily dose [RR = 1.06; 95% CI (0.79, 1.41)] (Table 8).

The details of the forest plots are annexed (S1 Fig) and additional effect estimates extracted from included studies are also annexed (S2 Table).

Adherence to the IPT (or placebo) regimen could be assessed from seven studies. The studies reported similarity of TB incidence in adherent and non-adherent participants although the proportion of non-adherence varied from study to study. None of these studies described reasons for non-adherence or predictors of non-adherence. (Table 2)

## Discussion

IPT has been one of the four main strategies of TB prevention in both HIV positive and negative patients since the introduction of Isoniazid.^41,42,43^ The clinical trials included in this review demonstrated that IPT was generally efficacious to prevent TB disease. Nevertheless, the width of calculated prediction intervals indicates that IPT may not be a beneficial strategy in all populations. Further research is needed to explore sources of this heterogeneity. Despite an effect on development of TB disease, a limited reduction in all-cause mortality was found; Some of the studies may have been under-powered to detect an effect on mortality or limited follow-up time precluded assessment of a long term effect. From the subgroup analysis, it appears that the six-month 300 mg daily IPT regimen reduced the risk of TB with the lowest number of adverse events.

The results of this meta-analysis confirm that a positive TST is a very strong indicator for the potential benefit of IPT also in countries with high burden of TB.^41,44^ Patients with a positive TST experienced a relative risk of TB disease of RR = 0.48; 95% CI (0.29, 0.82) compared to untreated patients or treated with placebo. For subjects with negative TST reactions, there was a moderate, but not statistically significant, protective effect (RR = 0.79; 95% CI (0.58, 1.08) compared to untreated patients. This is in agreement with previous reports.^31,44^ As TST reactivity also demonstrates a functional anti-mycobacterial response, a TST negative status can indicate either a lack of TB exposure, or result from severe immune deficiency with or without underlying latent TB. A TST positive status hence indicates a preserved immune response and this could affect the efficacy of IPT.^45^

ART contributes to the prevention of TB in HIV positive patients by maintaining the patient’s immune system.[45] Only two of the included studies (Rangaka *et*.*al*. and TEMPRANO) assessed the combined effect of ART and IPT on TB, all-cause mortality, or HIV disease progression and found that ART decreased the risk of all-types of TB and increased the risk of ADR. Further evidence from observational cohort studies indicate that antiretroviral therapy may significantly reduce the risk of TB.[28], [46–52] Despite the protective effects of ART, long-term incidence rates of TB remain high in people with HIV.[53–55] This is arguably related to suboptimal adherence,[56] the limited CD4-cell count recovery[57], persisting defects in TB-specific immune responses[58], or a high rate of re-exposure to infectious TB.[59] Even in patients who achieve CD4-cell counts above 500×10⁶/L TB risk remains about two times higher than background.[57] Recent clinical trials[60] and observational studies[26] reported that combined IPT and ART reduced the risk of TB and advanced HIV disease events and IPT achieved an additional TB disease risk reduction in patients on ART. Therefore, clinical trials or well-designed observational studies are needed to assess the TB prevention interventions in addition to ART. The benefit of secondary prophylaxis of IPT should ideally be investigated in high TB burden countries.

The potential benefit of IPT on the prevention of progression of immunodeficiency (HIV disease progression) in TST positives was only assessed in a single study with a RR = 0.36; 95% CI (0.15, 0.85). In TST negatives, however, the effect of IPT is nominal on HIV disease progression [RR = 1.00; 95% CI (0.88, 1.15)]. Data from a meta-analysis of randomized controlled trials of 4136 HIV patients previously demonstrated that IPT did not prevent progression of immunodeficiency.[61]

IPT did not appear to affect all-cause mortality in the total population studied, nor in any of the subgroups based on TST status, which is consistent with findings of other studies.[60] The pooled estimates did not reveal an association between IPT and adverse drug reactions.[61] However, higher dosage and duration was associated with a higher risk of the adverse drug reaction.

This review bears the following strengths. First, participants were stratified based on their TST status, which allowed identification of the subgroups that may benefit most from IPT. In contrast to previous systematic reviews, we assessed HIV disease progression and all-cause mortality across the different TST, ART exposure, IPT dose, and duration strata. Second, this review includes a recent clinical trial assessing the effect of IPT in ART patients which has substantial implications to the contemporary TB/HIV clinical practice of high ART adoption rate. Finally, given the high degree of clinical heterogeneity, the current review did not only present pooled effect estimates that were adjusted for between-study heterogeneity, but also calculated prediction intervals reflecting the expected range of the IPT efficacy in future studies with similar characteristics as those included in this meta-analysis. These intervals suggest that, despite the overall protective effect of IPT in HIV patients, non-beneficial effects may still be expected in some specific TST negative and TST unknown populations for yet unidentified reasons.

This review has also some limitations. A major limitation emanates from the small sample size of participants in TST positives and TST unknowns group rendering the pooled effect size of IPT across different outcomes rather imprecise. Furthermore, only one study assessed the combined effect of IPT with ART on prevention of TB, HIV disease progression and all-cause mortality, so no synthesis could be applied for this subgroup of patients. Despite an anticipated risk of isoniazid-resistant TB after inadvertent isoniazid monotherapy in high TB burden countries,[62] only one study assessed this effect of IPT and found an insignificant risk of drug-resistant TB. Hence, evaluating the risk of drug-resistant TB is a compelling area for future research. This review included manuscript available in English language only. Finally, collecting subject-level data from the included studies and performing an individual participant data meta-analysis could help to reduce the extent of the observed heterogeneity (e.g. by harmonizing the analysis of primary studies and accounting for differences in follow-up), and to analyze the presence of effect modification more thoroughly.[63], [64]

## Conclusions

This meta-analysis suggests a protective effect of IPT on development of TB in HIV infected patients, where TST positive patients benefit to a greater extent than patients with a negative TST. The effects on mortality and HIV disease progression were modest but there was some evidence indicating an additional benefit of IPT over ART alone in the prevention of TB disease. Future studies are necessary to determine the risk of drug-resistant TB after IPT use.

## Supporting Information

S1 Fig List of forest plots showing IPT effect on different outcomes and exposure strata. (PDF)

S1 Table A predefined table for data extraction from included studies. (DOCX)

S2 Table Additional effect estimates extracted from included studies. (DOCX)

S3 Table PRISMA 2009 checklist. (DOC)

S1 Text List of included studies. (DOCX)

S2 Text List of excluded studies. (DOCX)
