# Supplementary material for: Isoniazid Prophylactic Therapy for the Prevention of Tuberculosis in HIV Infected Adults: A Systematic Review and Meta-Analysis of Randomized Trials
Source: PLoS One. 2015 Nov 9;10(11):e0142290. doi: 10.1371/journal.pone.0142290 (PMC4638336; doi:10.1371/journal.pone.0142290)
Supplement: S1 Fig — (PDF) [file pone.0142290.s001.pdf]

1. Effect of IPT on all-types of TB in people with HIV

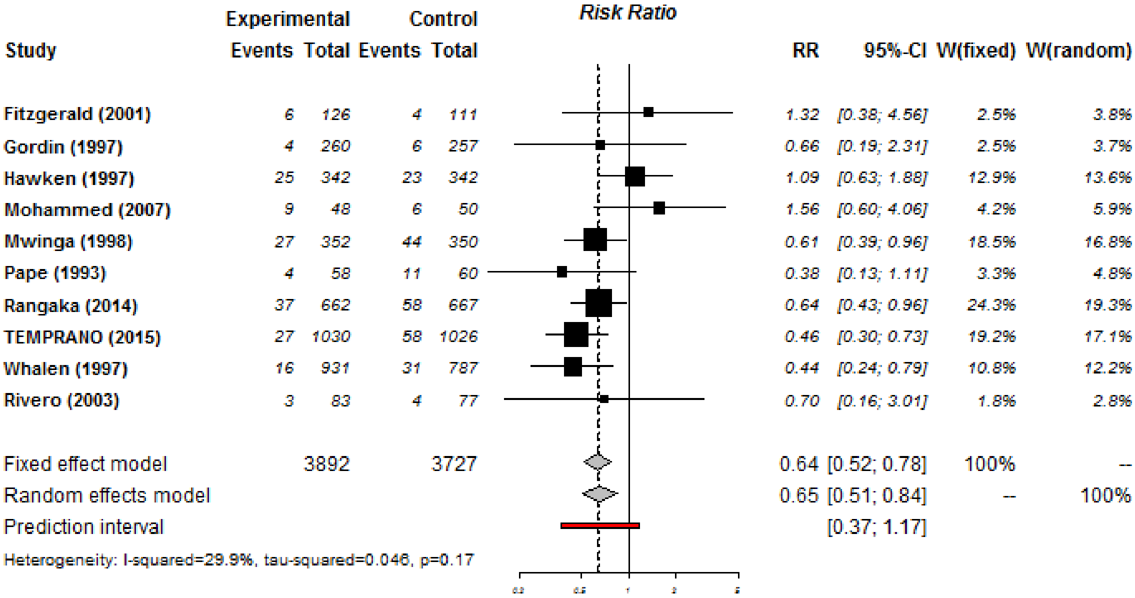

Study	Experimental Events	Experimental Total	Control Events	Control Total	RR	95%-CI	W(fixed)	W(random)
Fitzgerald (2001)	6	126	4	111	1.32	[0.38; 4.56]	2.5%	3.8%
Gordin (1997)	4	260	6	257	0.66	[0.19; 2.31]	2.5%	3.7%
Hawken (1997)	25	342	23	342	1.09	[0.63; 1.88]	12.9%	13.6%
Mohammed (2007)	9	48	6	50	1.56	[0.60; 4.06]	4.2%	5.9%
Mwinga (1998)	27	352	44	350	0.61	[0.39; 0.96]	18.5%	16.8%
Pape (1993)	4	58	11	60	0.38	[0.13; 1.11]	3.3%	4.8%
Rangaka (2014)	37	662	58	667	0.64	[0.43; 0.96]	24.3%	19.3%
TEMPRANO (2015)	27	1030	58	1026	0.46	[0.30; 0.73]	19.2%	17.1%
Whalen (1997)	16	931	31	787	0.44	[0.24; 0.79]	10.8%	12.2%
Rivero (2003)	3	83	4	77	0.70	[0.16; 3.01]	1.8%	2.8%
Fixed effect model		3892		3727	0.64	[0.52; 0.78]	100%	--
Random effects model					0.65	[0.51; 0.84]	--	100%
Prediction interval						[0.37; 1.17]		

Heterogeneity: I-squared=29.9%, tau-squared=0.046, p=0.17

1.1 Effect of isoniazid preventive therapy in all-types of tuberculosis in all patients with HIV infection

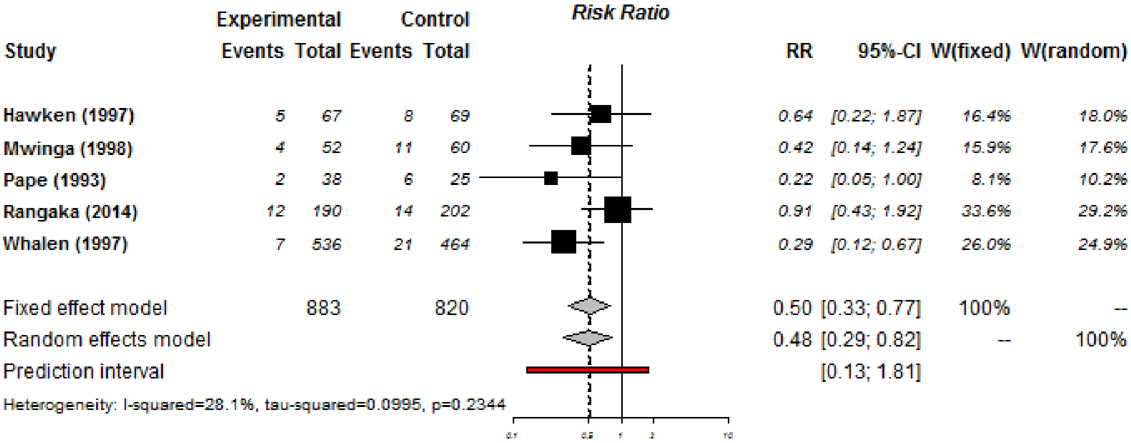

Study	Experimental Events	Experimental Total	Control Events	Control Total	RR	95%-CI	W(fixed)	W(random)
Hawken (1997)	5	67	8	69	0.64	[0.22; 1.87]	16.4%	18.0%
Mwinga (1998)	4	52	11	60	0.42	[0.14; 1.24]	15.9%	17.6%
Pape (1993)	2	38	6	25	0.22	[0.05; 1.00]	8.1%	10.2%
Rangaka (2014)	12	190	14	202	0.91	[0.43; 1.92]	33.6%	29.2%
Whalen (1997)	7	536	21	464	0.29	[0.12; 0.67]	26.0%	24.9%
Fixed effect model		883		820	0.50	[0.33; 0.77]	100%	--
Random effects model					0.48	[0.29; 0.82]	--	100%
Prediction interval						[0.13; 1.81]		

Heterogeneity: I-squared=28.1%, tau-squared=0.0995, p=0.2344

1.2 Effect of isoniazid preventive therapy in all-types of tuberculosis in TST positive patients with HIV infection

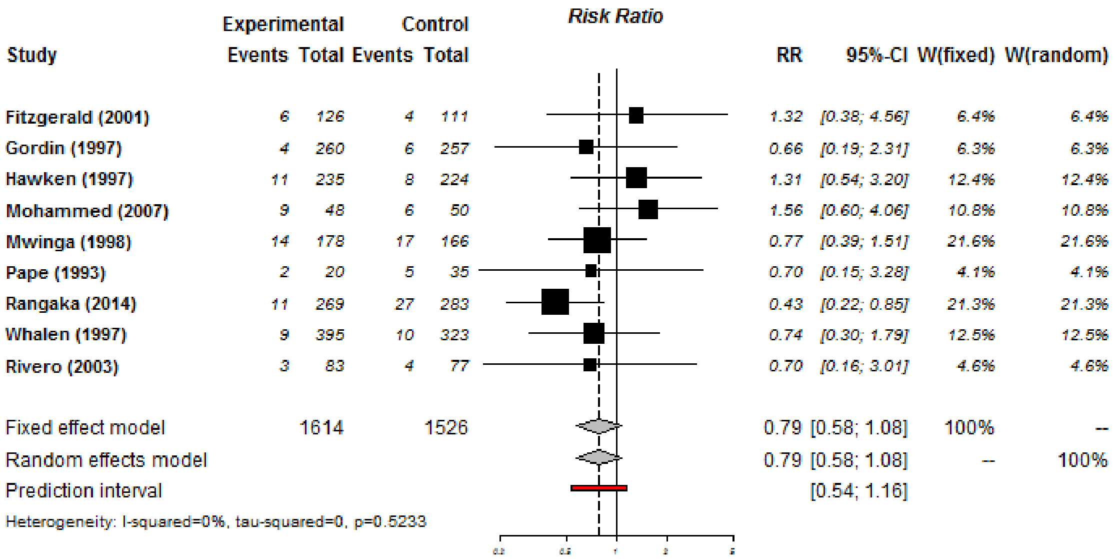

1.3 Effect of isoniazid preventive therapy on all-types of tuberculosis in TST negatives with HIV infection

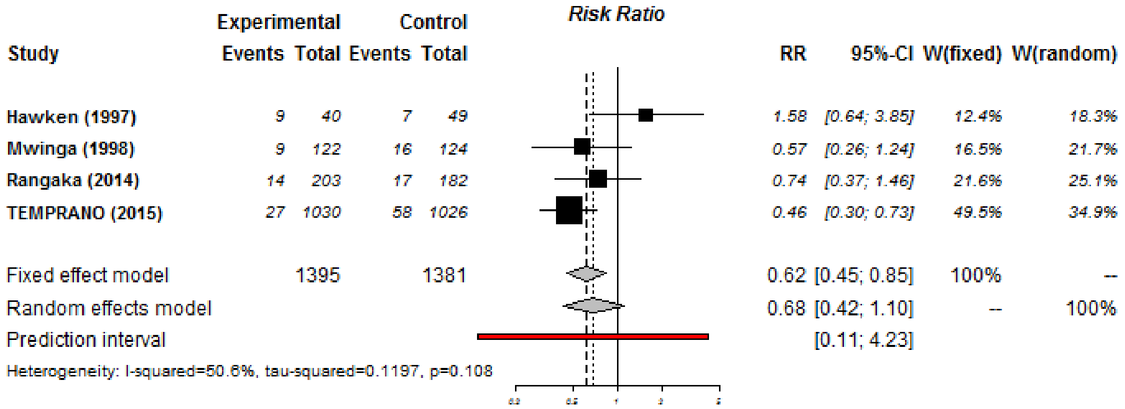

1.4 Effect of isoniazid preventive therapy on all-types of tuberculosis in TST unknowns with HIV infection

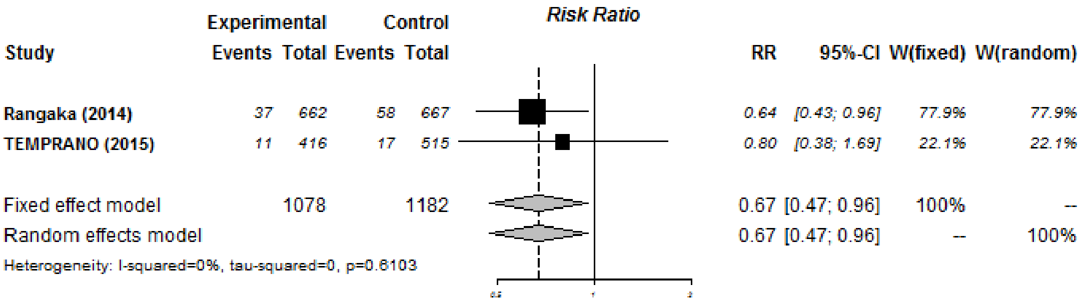

1.5. Effect of isoniazid preventive therapy on all-types of tuberculosis in patients with HIV infection receiving ART

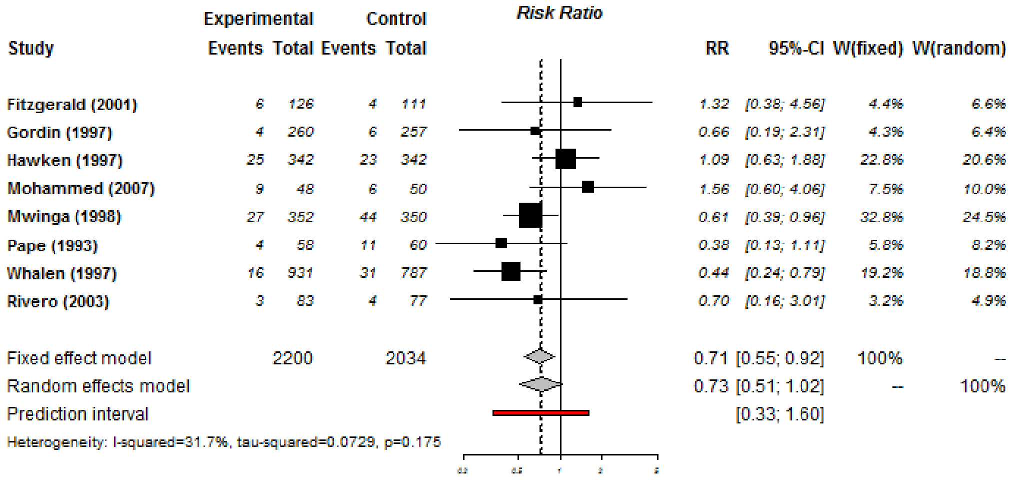

1.6 Effect of isoniazid preventive therapy on all-types of tuberculosis in patients with HIV infection without ART

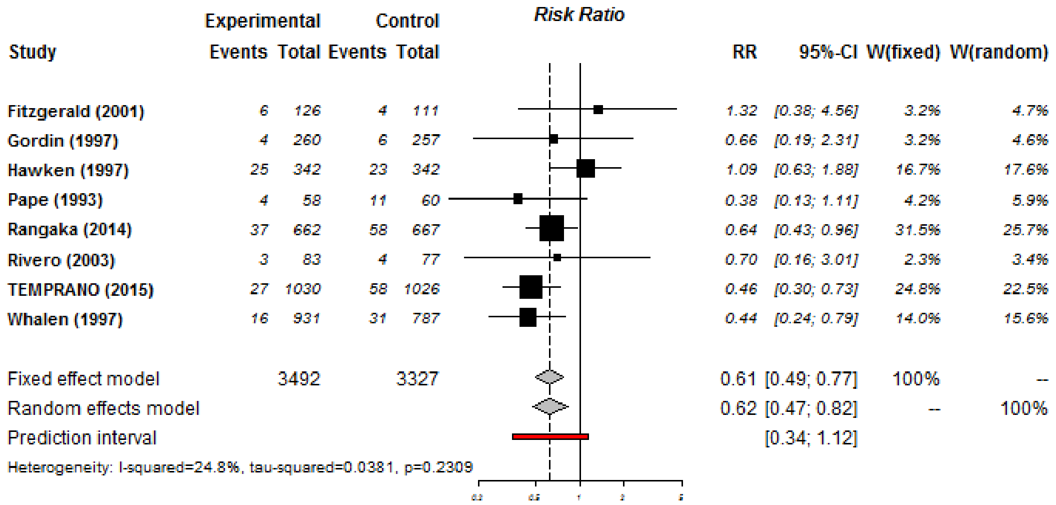

1.7 Effect of isoniazid preventive therapy on all-types of tuberculosis in patients with HIV infection and receiving 300mg isoniazid preventive therapy

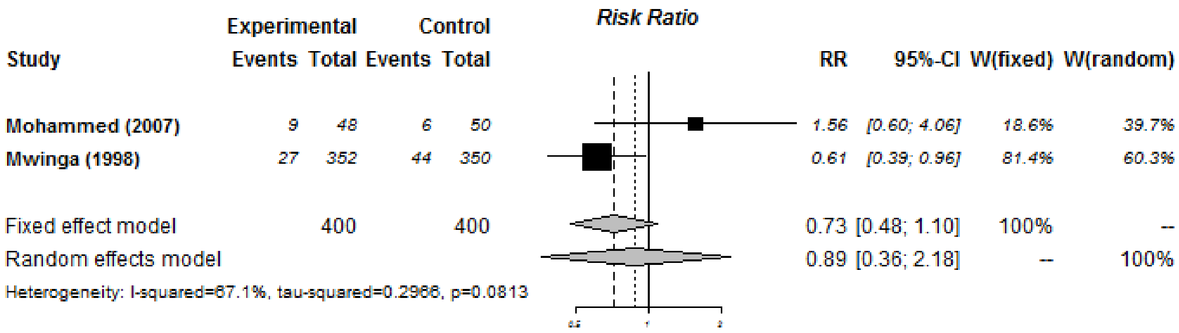

1.8 Effect of isoniazid preventive therapy on all-types of tuberculosis in patients with HIV infection receiving 900mg of isoniazid preventive therapy

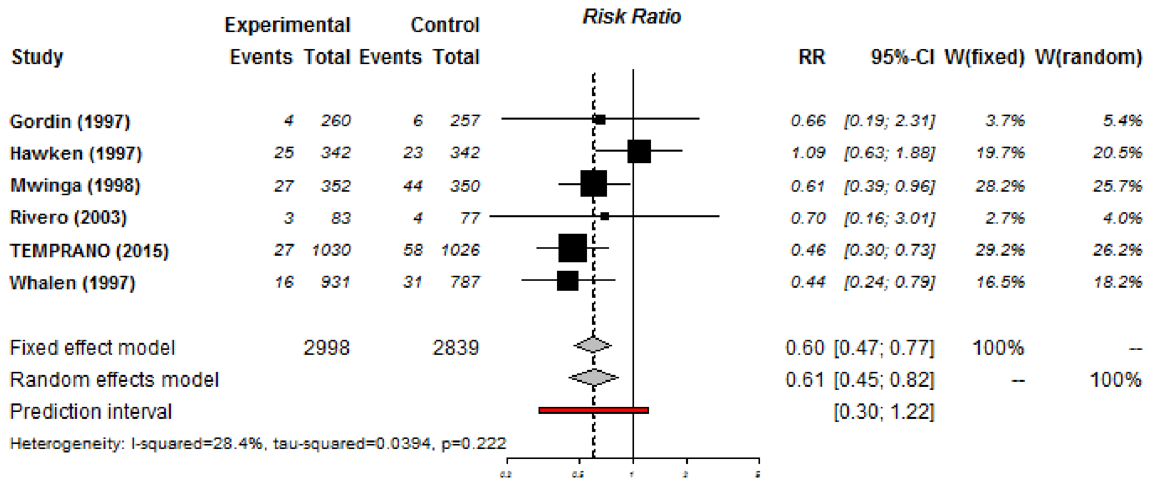

1.9 Effect of 6 months isoniazid preventive therapy on all-types of tuberculosis in patients with HIV infection

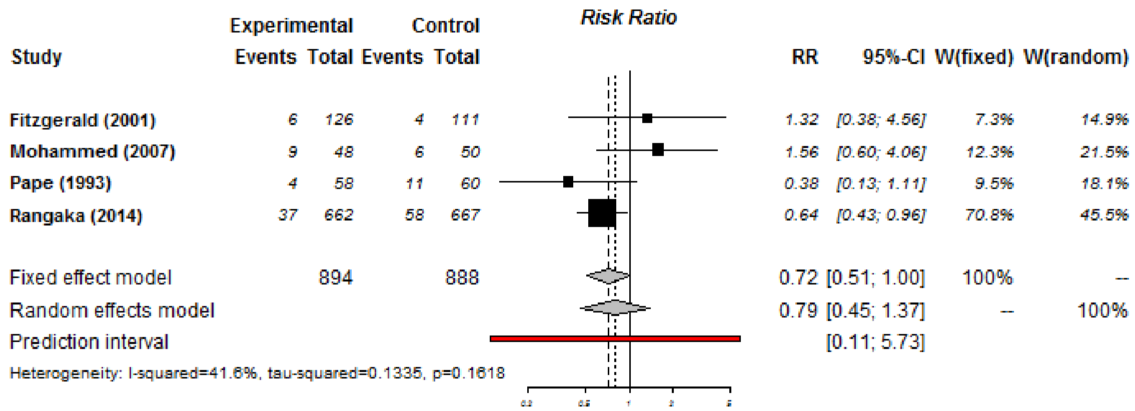

1.10 Effect of 12 months isoniazid preventive therapy on all-types of tuberculosis in patients with HIV infection

2. Effect of isoniazid preventive therapy in confirmed tuberculosis in patients with HIV infection

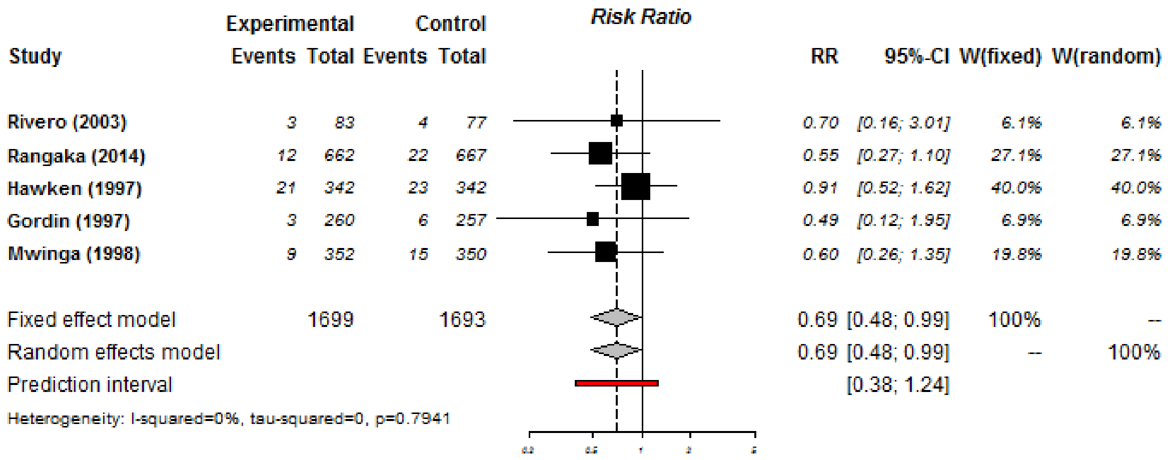

2.1 Effect of isoniazid preventive therapy in confirmed tuberculosis in all patients with HIV infection

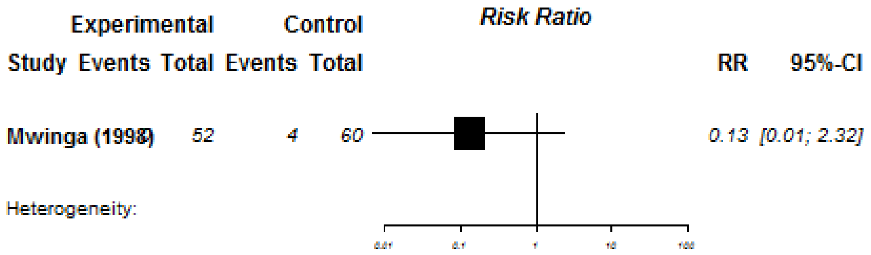

2.2 Effect of isoniazid preventive therapy in confirmed tuberculosis in TST positive patients with HIV infection

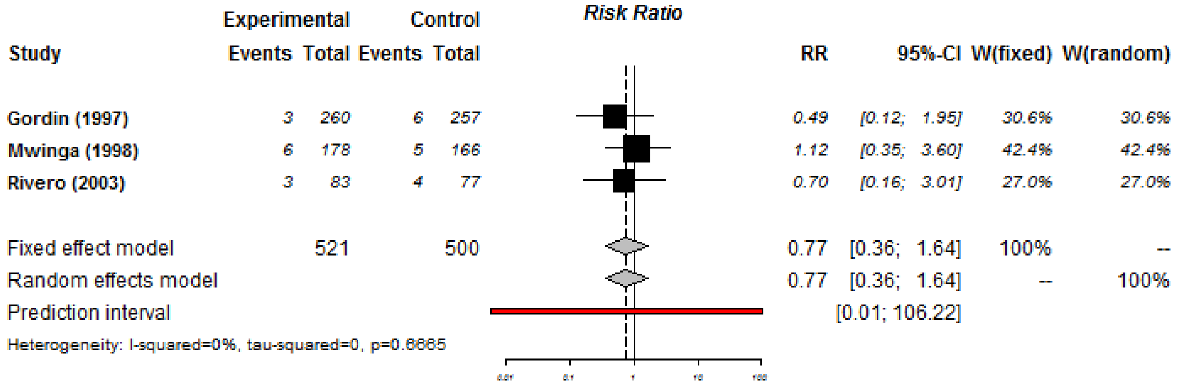

2.3 Effect of isoniazid preventive therapy on confirmed tuberculosis in TST negatives with HIV infection

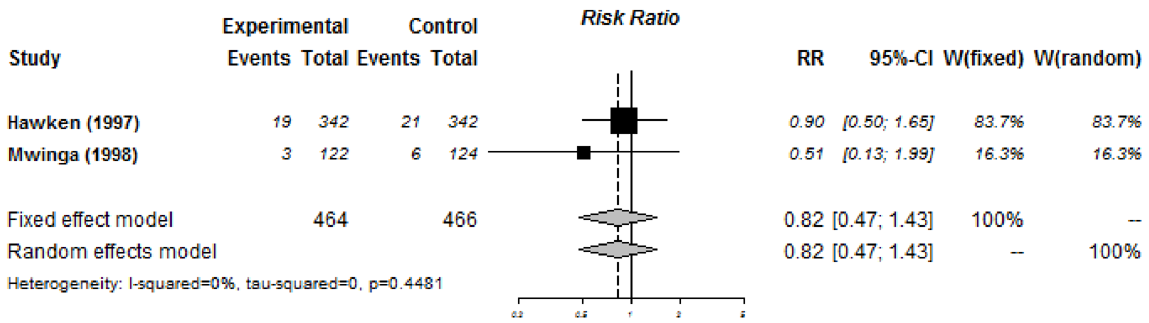

2.4 Effect of isoniazid preventive therapy on confirmed tuberculosis in TST unknowns with HIV infection

3. Effect of isoniazid preventive therapy in all-cause mortality in patients with HIV infection

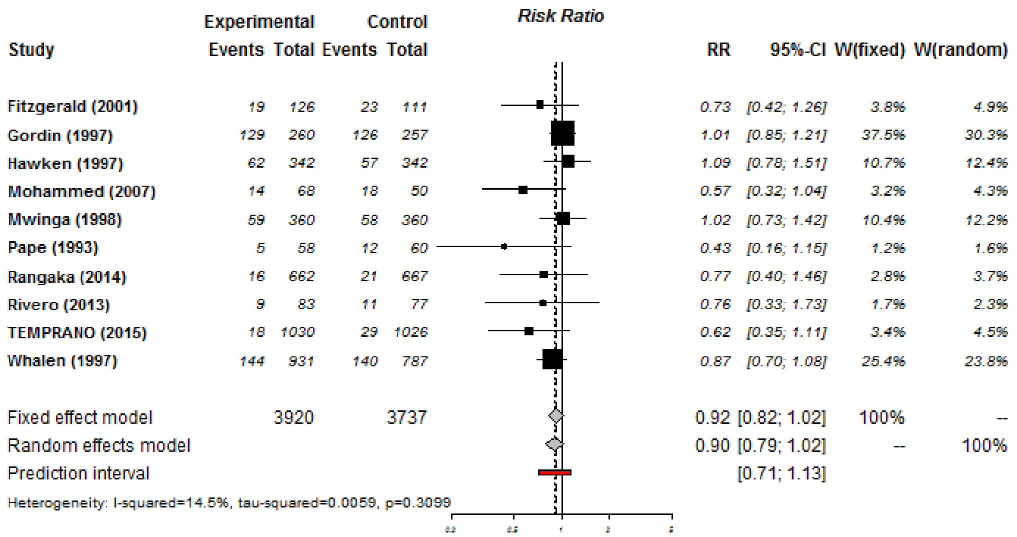

3.1 Effect of isoniazid preventive therapy in all-cause mortality in all patients with HIV infection

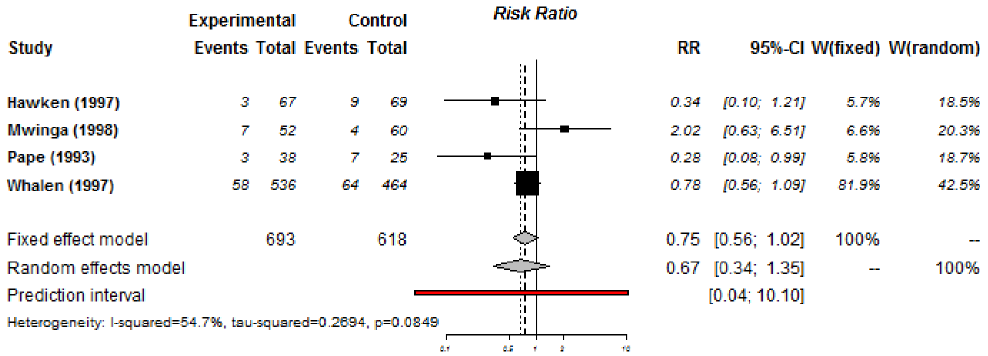

3.2 Effect of isoniazid preventive therapy in all-cause mortality in TST positive patients with HIV infection

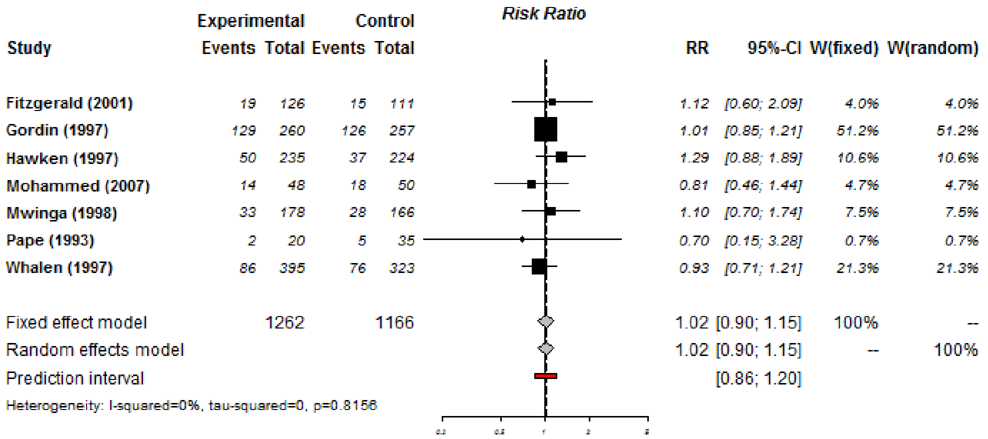

3.3 Effect of isoniazid preventive therapy on all-cause mortality in TST negatives with HIV infection

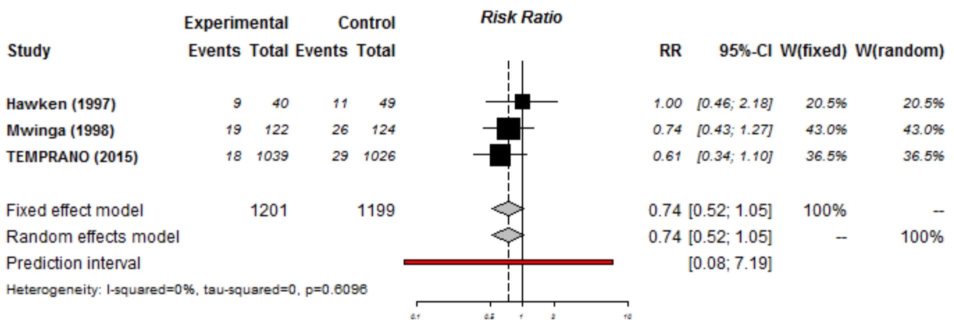

3.4 Effect of isoniazid preventive therapy on all-cause mortality in TST unknowns with HIV infection

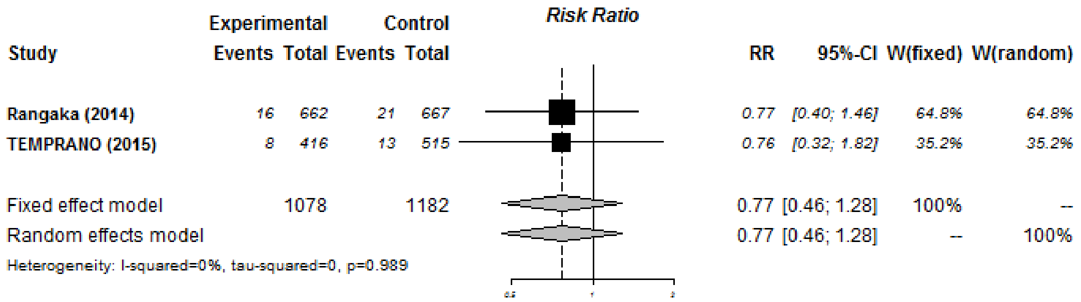

3.5 Effect of isoniazid preventive therapy on all-cause mortality in patients with HIV infection receiving ART

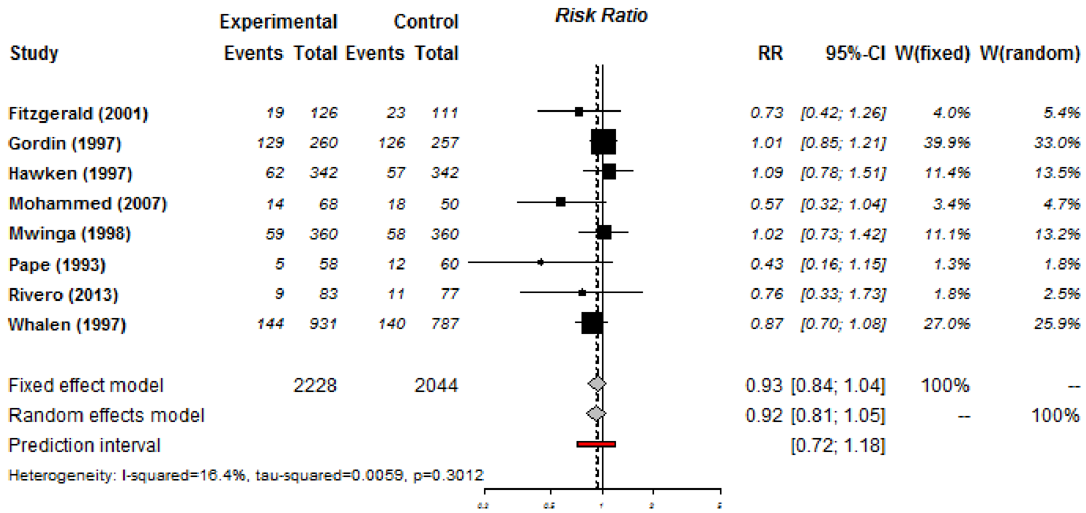

3.6 Effect of isoniazid preventive therapy on all-cause mortality in patients with HIV infection without ART

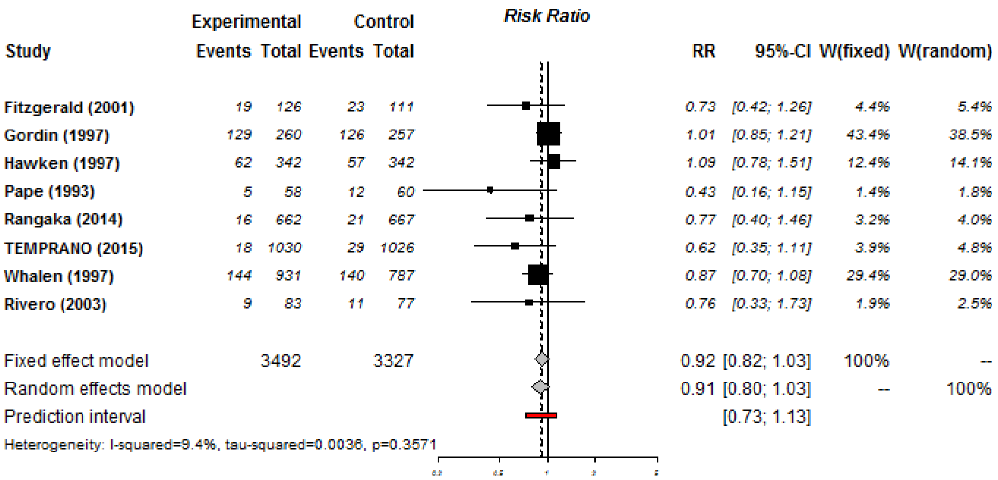

3.7 Effect of 300mg isoniazid preventive therapy on all-cause mortality in patients with HIV infection

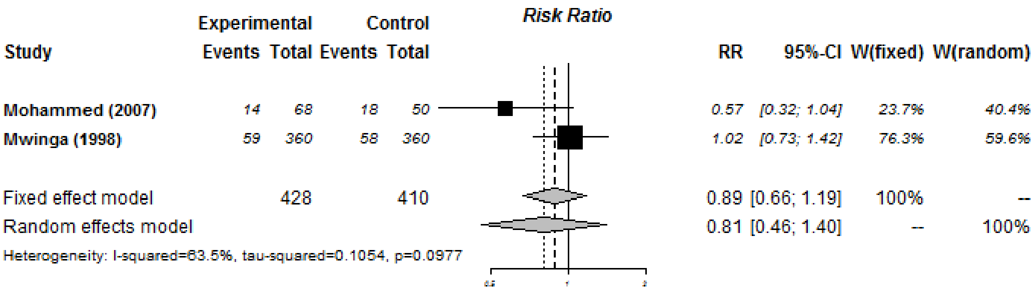

3.8 Effect of 900mg isoniazid preventive therapy on all-cause mortality in patients with HIV infection

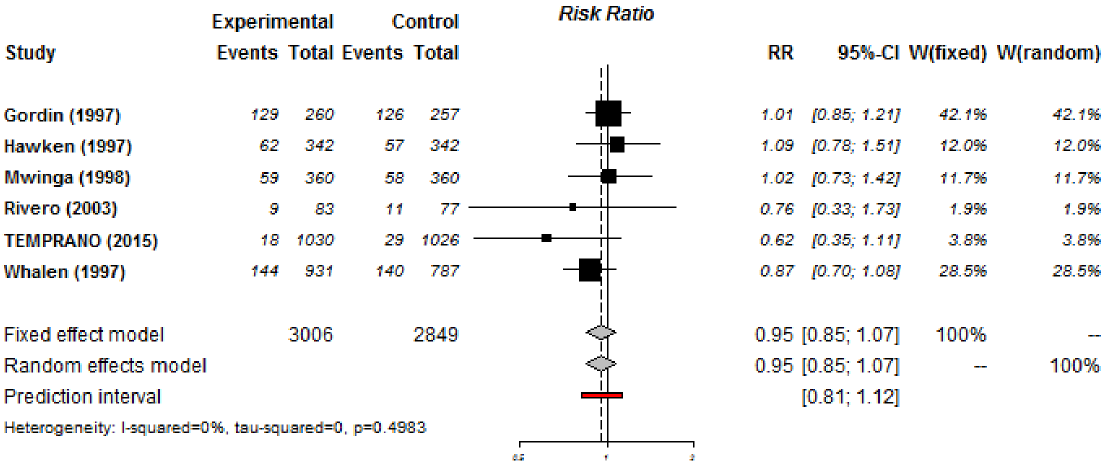

3.9 Effect of 6 months isoniazid preventive therapy on all-cause mortality in patients with HIV infection

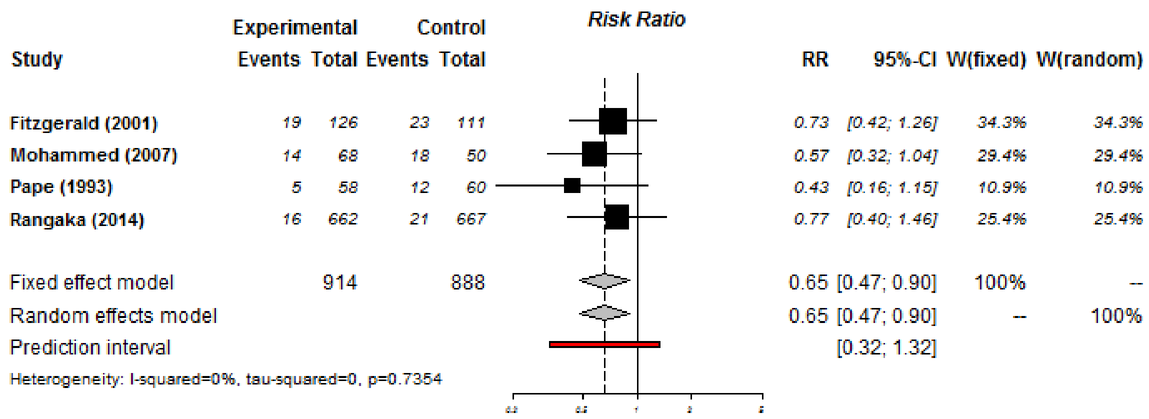

3.10 Effect of 12 months isoniazid preventive therapy on all-cause mortality in patients with HIV infection

4. Effect of isoniazid preventive therapy on HIV disease progression

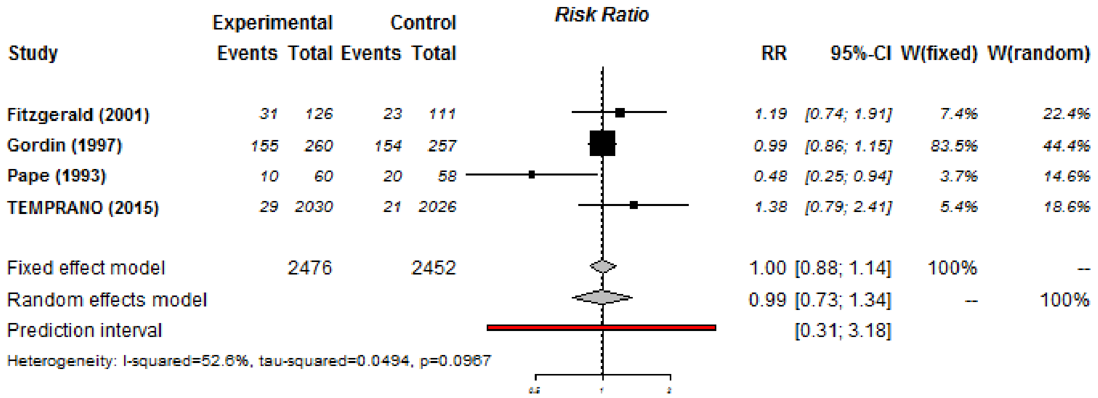

4.1. Effect of isoniazid preventive therapy on HIV disease progression in all patients

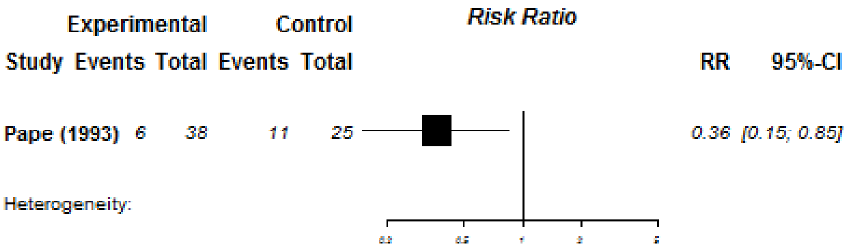

4.2. Effect of isoniazid preventive therapy on HIV disease progression in TST positive patients

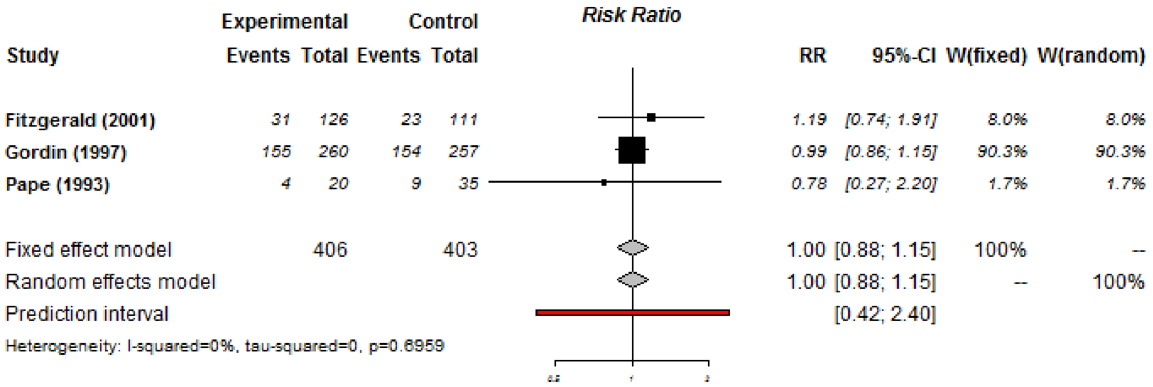

4.3 Effect of isoniazid preventive therapy on HIV disease progression in TST negatives

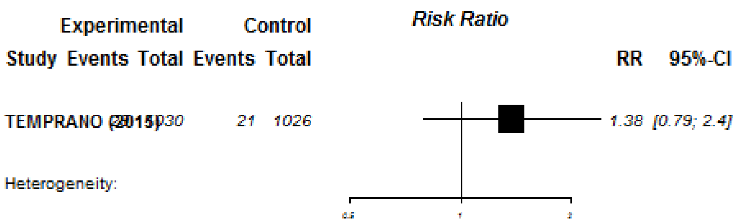

4.4 *Effect of isoniazid preventive therapy on HIV disease progression in TST unknowns*

5. Effect of isoniazid preventive therapy on adverse drug reaction

Study	Experimental Events	Experimental Total	Control Events	Control Total	RR	95%-CI	W(fixed)	W(random)
Gordin (1997)	24	260	24	257	0.99	[0.58; 1.69]	11.4%	20.1%
Hawken (1997)	11	342	5	342	2.20	[0.77; 6.26]	3.0%	8.7%
Mwinga (1998)	12	352	3	350	3.98	[1.13; 13.97]	2.1%	6.5%
Pape (1993)	0	58	0	60			0.0%	0.0%
Rangaka (2014)	102	662	94	667	1.09	[0.84; 1.42]	49.4%	31.7%
Rivero (2003)	6	83	0	77	12.07	[0.69; 210.64]	0.4%	1.5%
TEMPRANO (2015)	65	1030	79	1026	0.82	[0.60; 1.12]	33.0%	29.2%
Whalen (1997 TST positive	3	536	1	464	2.60	[0.27; 24.88]	0.6%	2.3%
Whalen (1997 TST negative	0	395	0	323			0.0%	0.0%
Fixed effect model		3718		3566	1.05	[0.87; 1.26]	100%	--
Random effects model					1.20	[0.85; 1.71]	--	100%
Prediction interval						[0.50; 2.88]		

Heterogeneity: I-squared=50.6%, tau-squared=0.0837, p=0.0588

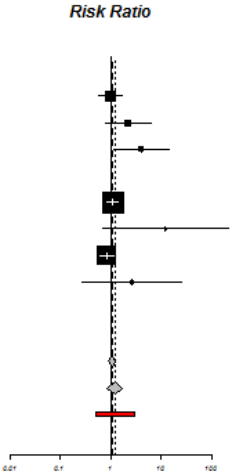

5.1. *Effect of isoniazid preventive therapy in adverse drug reaction in all patients*

Study	Experimental Events	Experimental Total	Control Events	Control Total	RR	95%-CI	W(fixed)	W(random)
Rangaka (2014)	102	662	94	667	1.09	[0.84; 1.42]	74.4%	55.1%
TEMPRANO (2015)	33	416	42	1026	1.94	[1.25; 3.01]	25.6%	44.9%
Fixed effect model		1078		1693	1.27	[1.01; 1.58]	100%	--
Random effects model					1.41	[0.81; 2.47]	--	100%

Heterogeneity: I-squared=79.2%, tau-squared=0.1297, p=0.0284

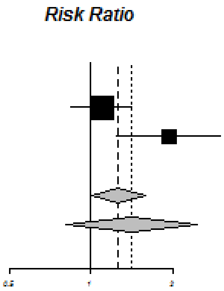

5.2. *Effect of isoniazid preventive therapy on adverse drug reaction in patients receiving ART*

Study	Experimental Events	Experimental Total	Control Events	Control Total	RR	95%-CI	W(fixed)	W(random)
Gordin (1997)	24	260	24	257	0.99	[0.58; 1.69]	64.9%	38.8%
Hawken (1997)	11	342	5	342	2.20	[0.77; 6.26]	17.2%	25.2%
Mwinga (1998)	12	352	3	350	3.98	[1.13; 13.97]	11.9%	20.8%
Pape (1993)	0	58	0	60			0.0%	0.0%
Rivero (2003)	6	83	0	77	12.07	[0.69; 210.64]	2.3%	6.2%
Whalen (1997 TST positive	3	536	1	464	2.60	[0.27; 24.88]	3.7%	9.1%
Whalen (1997 TST negative	0	395	0	323			0.0%	0.0%
Fixed effect model		2026		1873	1.47	[0.95; 2.27]	100%	--
Random effects model					2.06	[0.96; 4.40]	--	100%
Prediction interval						[0.24; 17.88]		

Heterogeneity: I-squared=45.9%, tau-squared=0.3116, p=0.1167

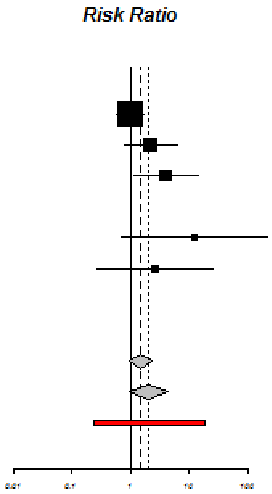

5.3 *Effect of isoniazid preventive therapy on adverse drug reaction in patients without ART*

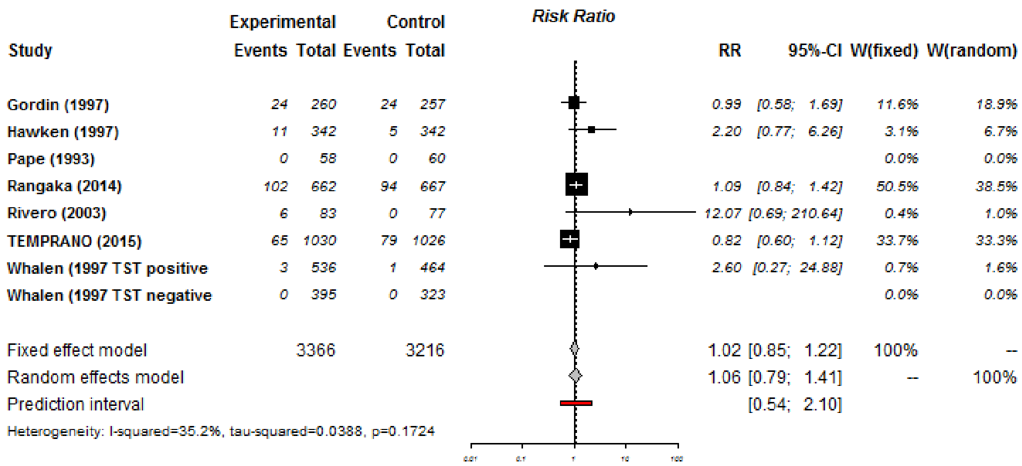

5.4 Effect of 300mg isoniazid preventive therapy on adverse drug reaction in patients with HIV infection

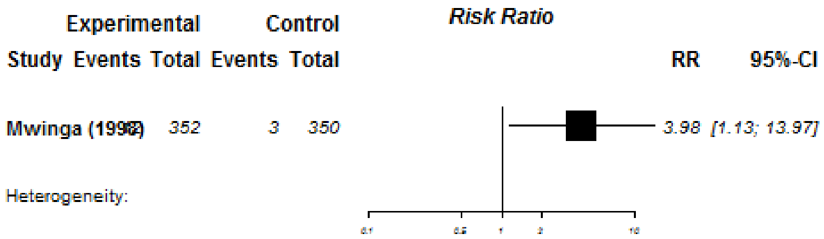

5.5 Effect of 900mg isoniazid preventive therapy on adverse drug reaction in patients with HIV infection

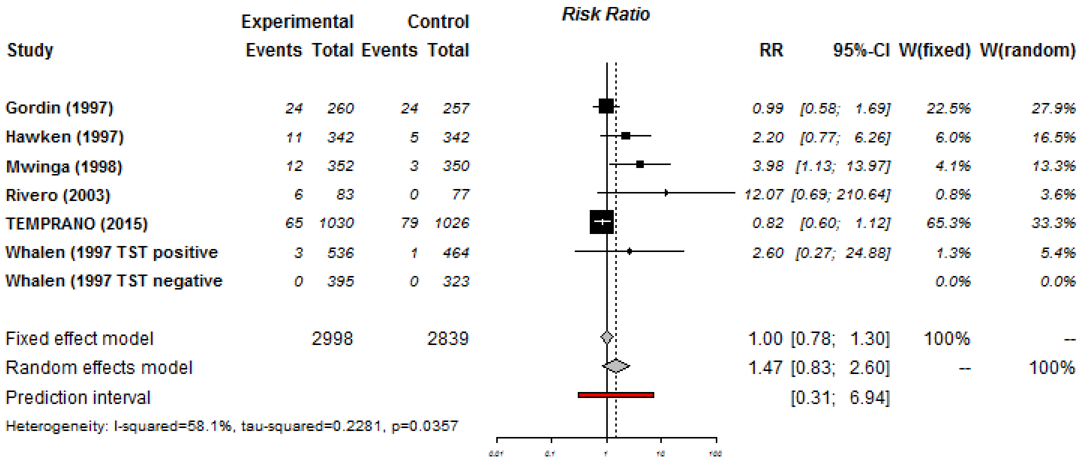

5.6 Effect of 6 months isoniazid preventive therapy on adverse drug reaction in patients with HIV infection

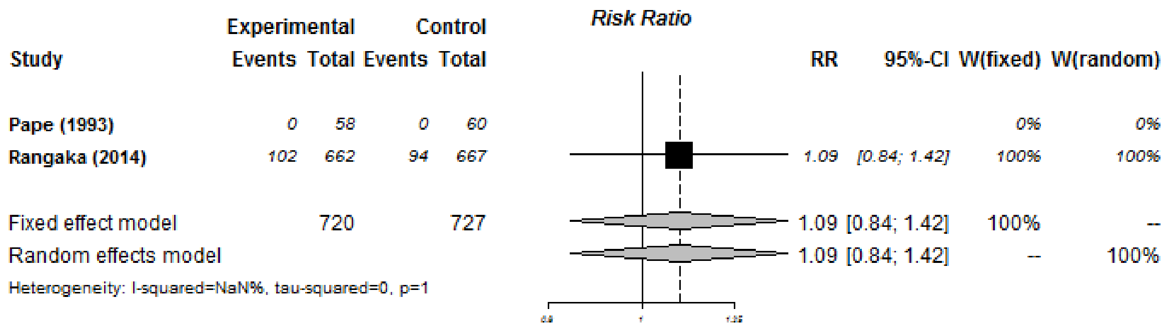

5.7 Effect of 12 months isoniazid preventive therapy on adverse drug reaction in patients with HIV infection
