# Supplementary material for: Isoniazid Prophylactic Therapy for the Prevention of Tuberculosis in HIV Infected Adults: A Systematic Review and Meta-Analysis of Randomized Trials
Source: PLoS One. 2015 Nov 9;10(11):e0142290. doi: 10.1371/journal.pone.0142290 (PMC4638336; doi:10.1371/journal.pone.0142290)
Supplement: S1 Table — (DOCX) [file pone.0142290.s002.docx]

**Appendix 1. Data extraction table**

| **S. No** | **Characteristics** | **Extracted data (Description)** | |
| --- | --- | --- | --- |
| 1 | Authors, Study area, & years of publication |  | |
| 2 | Method (randomization) |  | |
| 3 | Domain:(Participants) |  | |
| 4 | Intervention (IPT and other preventive therapies ) |  | |
| 5 | TST test |  | |
| 6 | ART enrolment |  | |
| 7 | Outcome |  | |
| 8 | Relative risk estimates for each outcomes |  | |
| **Risk of bias** | | | |
|  | **Item** | **Authors Judgment** | **Description** |
| 1 | Allocation concealment |  |  |
| 2 | Blinding (All outcomes) |  |  |
| 3 | Incomplete outcome data (well addressed) |  |  |
| 4 | Selective reporting |  |  |
