# Supplementary material for: Isoniazid Prophylactic Therapy for the Prevention of Tuberculosis in HIV Infected Adults: A Systematic Review and Meta-Analysis of Randomized Trials
Source: PLoS One. 2015 Nov 9;10(11):e0142290. doi: 10.1371/journal.pone.0142290 (PMC4638336; doi:10.1371/journal.pone.0142290)
Supplement: S2 Table — (DOCX) [file pone.0142290.s003.docx]

**Appendix 2: Additional effect estimates extracted from the selected articles.**

| **Studies** | **Tuberculosis** | | | **Acquired Immunodeficiency Syndrome (AIDS)** | | | **All-cause mortality** | | | **Adverse drug reaction** |
| --- | --- | --- | --- | --- | --- | --- | --- | --- | --- | --- |
|  | **TST positive** | **TST negative** | **Total** | **TST positive** | **TST negative** | **Total** | **TST positive** | **TST negative** | **Total** | **Total** |
| Fitzgerald 2001 |  | RRu=1.26**^§^** |  |  | RRu=1.12**^§^** |  |  | RRu=1.05**^§^** |  |  |
| Gordin 1997 |  | RRu=0.44**^§^** |  |  | RRu=0.99**^§^** |  |  | RRu=0.99**^§^** |  | RR=0.99 |
| Hawken 1997 | RRu=0.69  HRa=0.60 | RRu=1.20  HRa*=1.23 | RRu=1.11  HRu=1.11**^§^**  HRa=0.92 |  |  |  | HRa=0.33 | HRa=1.39^*^ | RRu=1.11  HRa=1.08 | RR=2.2 |
| Mohammed 2007 |  | HRu=1.59  HRa=2.02 |  |  |  |  |  | RRu=2.99 |  |  |
| Mwinga 1998 | RRu=0.25**^§^**  RRu=0.13**^β^*** | RRu=0.83**^§^** | RRu=0.62 |  |  |  | RRu=2.15**^§^** | RRu=1.10**^§^** | RRu=1.05 | RRu=3.98 |
| Pape 1993 | RRu=0.17^*^**^§^** | RRu=0.56^*^**^§^** | RRu=0.29^*^**^§^** | RRu=0.36**^§*^** | RRu=0.62 | RRu=040 | RRu=0.22 | RRu=0.56 | RRu=0.34 |  |
| Rangaka 2014 | HRu=0.92  Hra=0.86 | Hru=0.41**  Hra=0.43** | Hru=0.63*** |  |  |  |  |  | Hru=0.72 | RR=1.66 |
| Rivero 2003 |  | RRu=1.07 |  |  |  |  |  | RRu=1.17 |  |  |
| TEMPRANO 2015 |  |  | RRu=0.44 |  |  |  |  |  | RRu=0.61 | RRu=0.86^α^  RRu=0.76^µ^ |
| Whalen 1997 | RRu=0.33  RRa=0.32 | RRu=0.75**  RRa=0.73** | RRu=0.49 |  |  |  | RRu=0.87**^§^** | RRu=1.05 | RR=0.97 |  |

**RRu:** Unadjusted relative risk ratio; **RRa**: Adjusted relative risk ratio; **HRu**: Unadjusted hazard ratio; **HRa:** Adjusted hazard ratio; **HR**: Hazrd ratios; ****** Anergy cohort; **^*^** statistically significant; **^§^** Rate ratio; **^β^** Confirmed Tuberculosis; **TST**: Tuberculin Skin Test; ^α^ <6 months after randomization; ^µ^ 6-30 months after randomization
