# Supplementary material for: Isoniazid Prophylactic Therapy for the Prevention of Tuberculosis in HIV Infected Adults: A Systematic Review and Meta-Analysis of Randomized Trials
Source: PLoS One. 2015 Nov 9;10(11):e0142290. doi: 10.1371/journal.pone.0142290 (PMC4638336; doi:10.1371/journal.pone.0142290)
Supplement: S1 Text — (DOCX) [file pone.0142290.s005.docx]

**Supplementary text 1. Included studies for the review**

1. Fitzgerald D W, Severe P, Joseph P, et al. No effect of isoniazid prophylaxis for purified protein derivative-negative HIV-infected adults living in a country with endemic tuberculosis: results of a randomized trial. J Acquir Immune Defic Syndr 2001; 28: 305–307.
2. Gordin FM, Matts JP, Miller C et al. A controlled trial of isoniazid in persons with anergy and human immunode®ciency virus infection who are at high risk for tuberculosis. N Engl J Med 1997, 337:315±320.
3. Hawken MP, et al. Isoniazid preventive therapy for tuberculosis in HIV-1-infected adults: Results of a randomized controlled trial. AIDS. 1997; 11 (7):875–882.
4. Mohammed, A.; Myer, L.; Ehrlich, R.; Wood, R.; Cilliers, F.; Maartens, G.; Randomised controlled trial of isoniazid preventive therapy in South African adults with advanced HIV disease; The International Journal of Tuberculosis and Lung Disease, Volume 11, Number 10, October 2007, pp. 1114-1120(7)
5. Mwinga A, Hosp M, Godfrey-Faussett P, et al. Twice weekly tuberculosis preventive therapy in HIV infection in Zambia. AIDS 1998; 12: 2447–57
6. Pape JW, Jean SS, Ho JL et al. Effect of isoniazid prophylaxis on incidence of active tuberculosis and progression of HIV infection. Lancet 1993, 342:268±272.
7. Rangaka et al; Isoniazid plus antiretroviral therapy to prevent tuberculosis: a randomised double-blind, placebo-controlled trial; the lancet 2014: DOI: 10.1016/S0140-6736(14) 60162-8
8. Whalen C.C., Johnson J.L., Okwera A., Hom D.L., Huebner R., Mugyenyi P. et al.; A trial of three regimens to prevent tuberculosis in Ugandan adults infected with the human immunodeficiency virus, Uganda-Case Western Reserve University Research Collaboration; N Engl J Med, 337 (1997), pp. 801–808
9. The TEMPRANO ANRS 12136 Study Group. A trial of early antiretrovirals and isoniazid preventive therapy in Africa. N Engl J Med 2015;373:808-822
10. Rivero A, Lopez-Cortes L, Castillo R, Lozano F, Gracia MA, Diez F, Escribano JC, Canueto J, Pasquau J, Hernandez JJ, Polo R, Martinez-Marcos FJ, Kindelan JM, Rey R. A randomized trial of three regimens to prevent tuberculosis in HIV-infected patients with anergy. Enferm Infecc Microbiol Clin 2003;21(6):287–292.

**Excluded studies**

1. Churchyard GJ, Fielding KL, Lewis JJ, et al. A trial of mass isoniazid preventive therapy for tuberculosis control. N Engl J Med 2014;370:301-310
2. Fitzgerald 2000: Fitzgerald DW, Desvarieux M, Severe P, Joseph P, Johnson WD, Pape JW. [Effect of post treatment isoniazid on prevention of recurrent tuberculosis in HIV–1 infected indivduals: a randomized trial]. The Lancet 2000;356: 1470–1474.
3. Garcia 1993: GraciaML, Valdespino JL, Garcia-SanchoC,Weissenbacher M, Daniels E, Peruga A, Palacios M, Loo E, Cruz C, LinaJL, Luna M, Mayar ME, Romero C, Pineda L, Sepulveda J. Compliance and side effects to chemoprophylaxis for TB in HIV+. Mexican experience. IX International Conference on AIDS. 1993; Vol. Abstract–Po–BO7–11.
4. Gordin 2000: Gordin F, Chaisson RE, Matts JP, et al.Rifampin and pyrazinamide vs isoniazid for prevention of tuberculosis in HIV-Infected persons. An international randomized trial. JAMA 2000;283:1445–1450.
5. Grant 2005: Grant AD, Charalambous S, Fielding KL, Day JH, Corbett EL, Chaisson RE, De Cock KM, Hayes RJ, Churchyard GJ. Effect of routine isoniazid preventive therapy on tuberculosis incidence among HIV-infected men in South Africa: a novel randomized incremental study. JAMA 2005; 293(22):2719–2725.
6. Halsey 1998: Halsey NE, Coberly JS, Desormeaux J, Losikoff P, Atkinson J, Moulton LH, ContaveM, Johnson M, Davis H, Geitre L, Johnson E, Huebner R, Boulos R, Chaisson R. Randomized trial of isoniazid versus rifampicine and pyrazinamide for prevention of tuberculosis in HIV-1 infection. The Lancet 1998;351(9105):786–792.
7. Lim HJ, Okwera A, Mayanja-Kizza H, Ellner JJ, Mugerwa RD, Whalen CC. Effect of Tuberculosis Preventive Therapy on HIV Disease Progression and Survival in HIV-Infected Adults. HIV clinical trials. 2006;7(4):172-183. doi:10.1310/hct0704-172.
8. Martinez 2000: Martinez AEM, Cuadra F, Solera J, Macia MA, Geijo P, Sanchez MPA, Rodriguez ZM, Largo J, Sepilveda MA, Rosa C, Sanchez L, Espinosa A, Mateos F, Blanch JJ. Evaluation of 2 tuberculosis chemoprohylaxis regimens in patients infected with human immunodeficiency virus. The Gecmei Group. Medicina Clinica (Barcelona) 2000;115(5): 161–165.
9. Martinson NA, Barnes GL, Moulton LH, et al. New regimens to prevent tuberculosis in adults with HIV infection. N Engl J Med 2011;365:11-20
10. Matteelli 1998: Matteelli A, Carosi G, Signorini L, Tebaldi A, Caligaris S, Gulletta M. Multinational study for preventive therapy of latent tuberculosis in HIV positive individuals: isoniazid vs. rifabutin. 12th World AIDS Conference. 1998; Vol. Abstract 60858.
11. Rivero 2007: Rivero A, López-Cortés L, Castillo R, Verdejo J, García MA, Martínez-Marcos FJ, Díez F, Escribano JC, Canueto J, Lozano F, Pasquau J, Hernández JJ, Márquez M, Kindelán JM. A Randomized clinical trial investigating three chemoprophylaxis regimens for latent tuberculosis infection in HIV-infected patients. Enferm Infecc Microbiol Clin 2007;25(5):305–310.
12. Saenghirunvatta 1996: Saenghirunvattana S. [Effect of isoniazid prophylaxis on incidence of active uberculosis among Thai HIV–infected individuals]. J. Med Association Thai 1996;79(5):285–287.
13. Souza, Claudia Teresa Vieira de et al. Effectiveness and safety of isoniazid chemoprophylaxis for HIV-1 infected patients from Rio de Janeiro. Mem. Inst. Oswaldo Cruz [online]. 2009, vol.104, n.3, pp. 462-467.
14. Sterling TR, Villarino ME, Borisov AS, et al. Three months of rifapentine and isoniazid for latent tuberculosis infection. N Engl J Med 2011;365:2155-2166
15. Swaminathan S, Menon PA, Gopalan N, Perumal V, Santhanakrishnan RK, et al. (2012) Efficacy of a Six-Month versus a 36-Month Regimen for Prevention of Tuberculosis in HIV-Infected Persons in India: A Randomized Clinical Trial. PLoS ONE 7(12): e47400. doi:10.1371/journal.pone.0047400.
